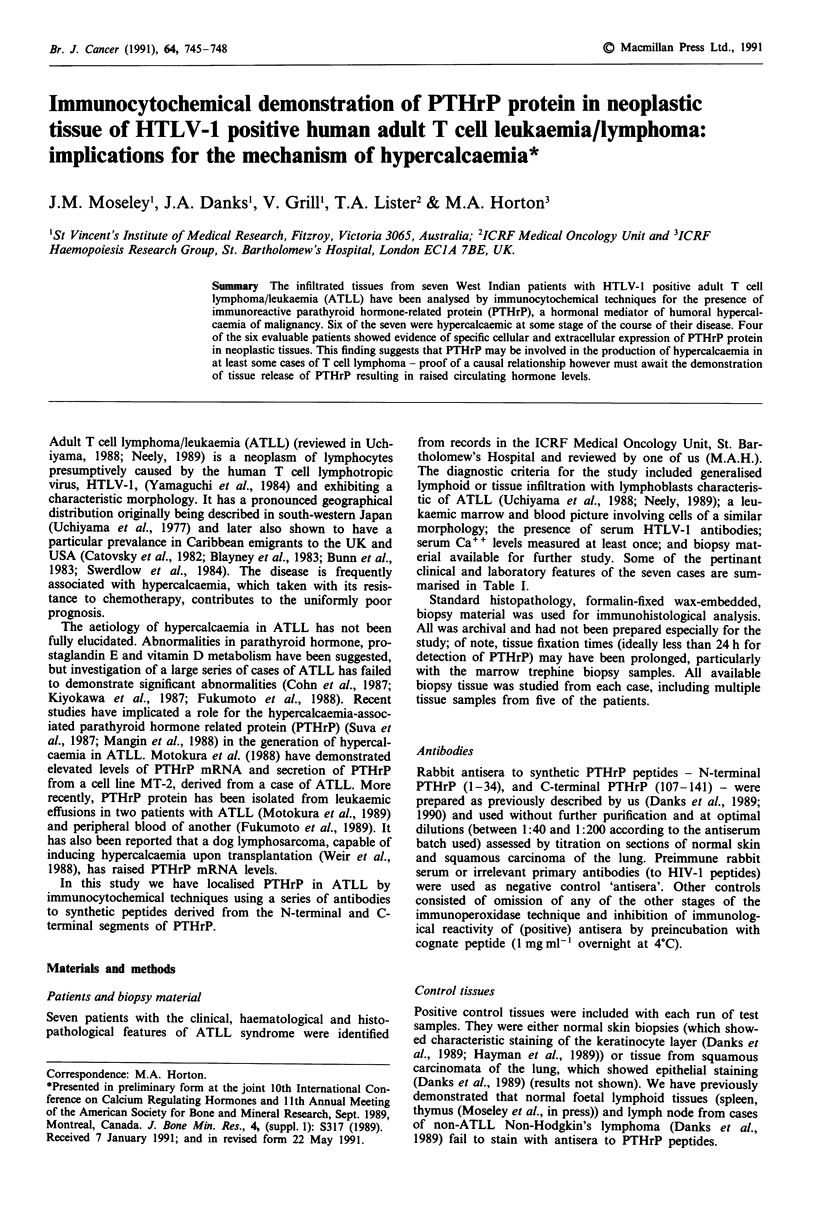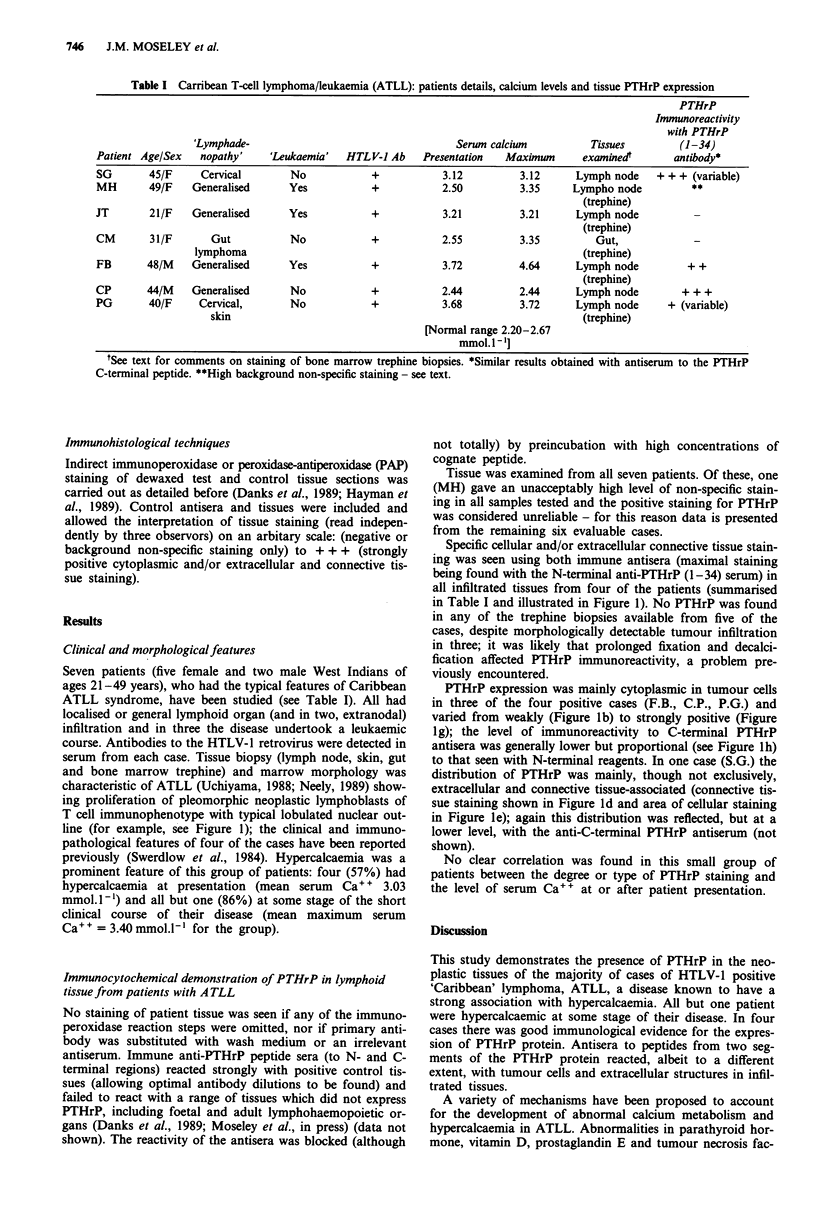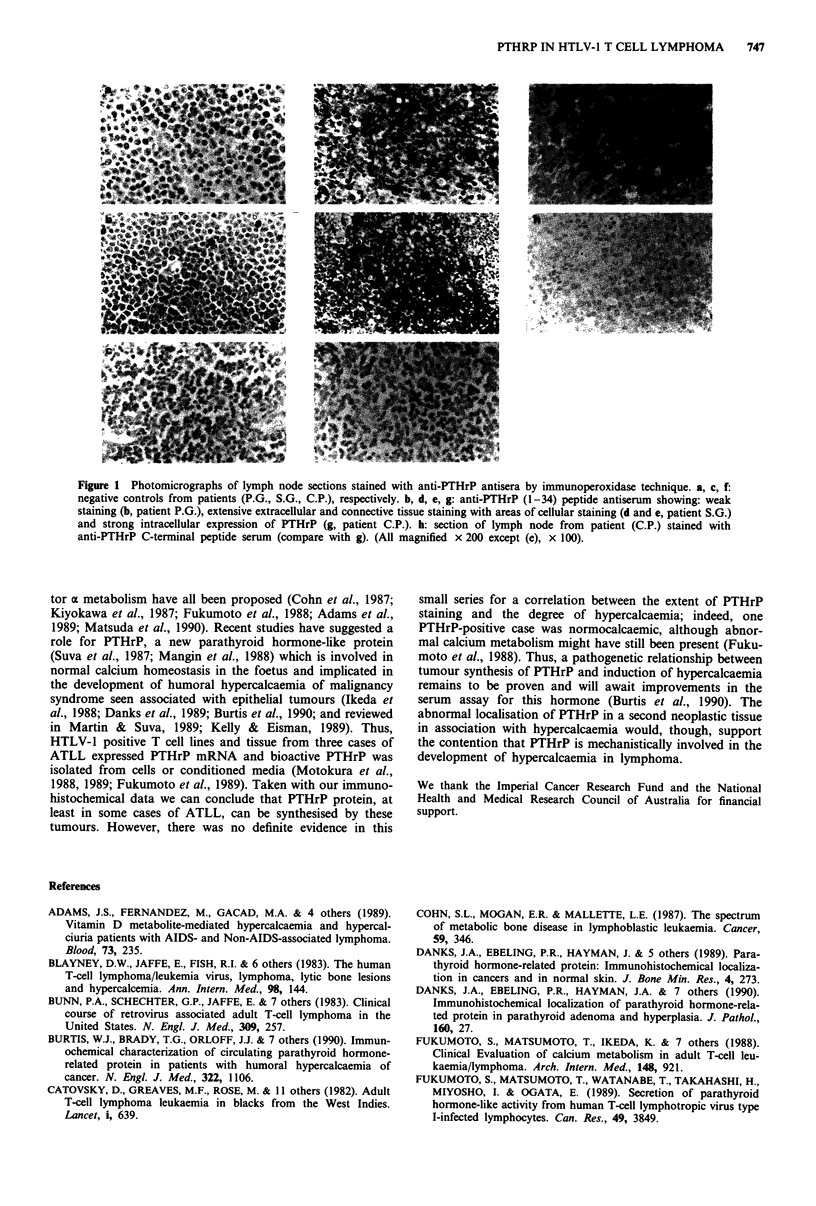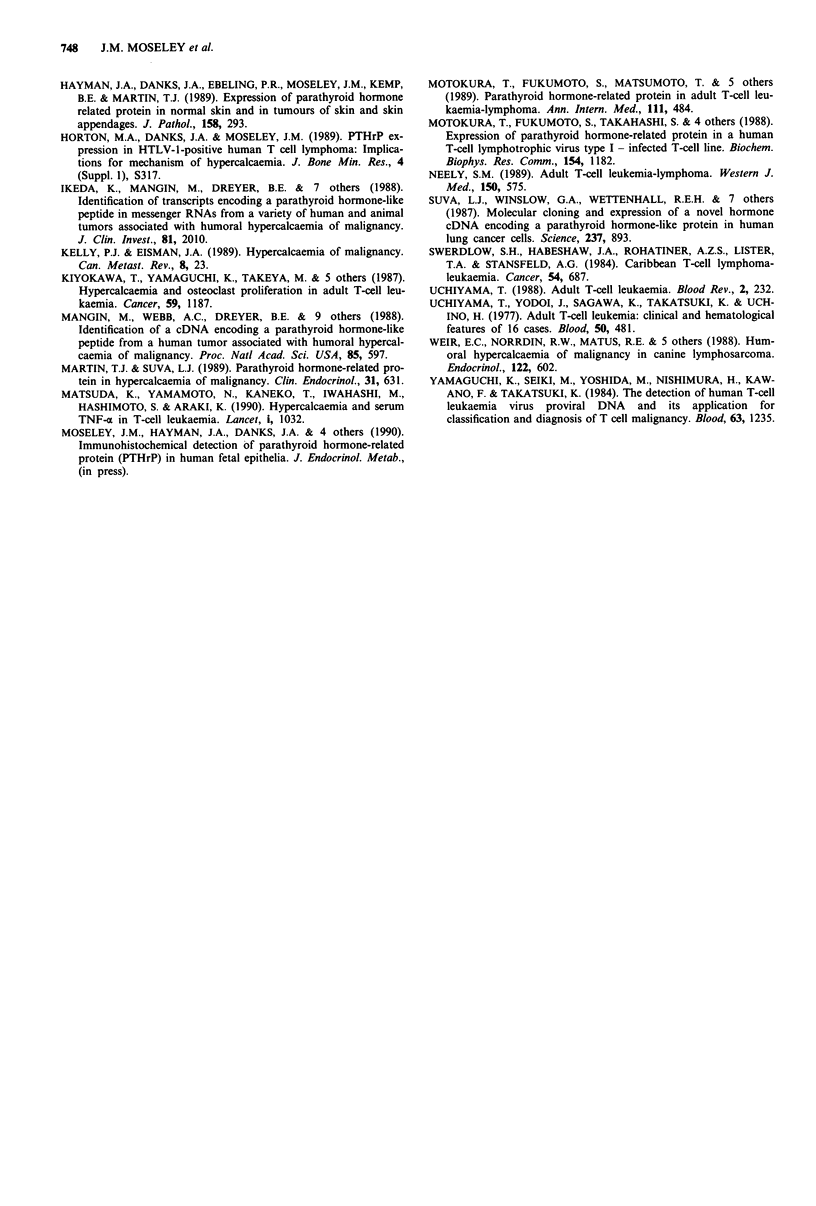# Immunocytochemical demonstration of PTHrP protein in neoplastic tissue of HTLV-1 positive human adult T cell leukaemia/lymphoma: implications for the mechanism of hypercalcaemia.

**DOI:** 10.1038/bjc.1991.391

**Published:** 1991-10

**Authors:** J. M. Moseley, J. A. Danks, V. Grill, T. A. Lister, M. A. Horton

**Affiliations:** St Vincent's Institute of Medical Research, Fitzroy, Victoria, Australia.

## Abstract

**Images:**


Br. J. Cancer (1991), 64, 745-748 © Macmillan Press Ltd., 1991

### Immunocytochemical demonstration of PTHrP protein in neoplastic tissue of HTLV-1 positive human adult T cell leukaemia/lymphoma: implications for the mechanism of hypercalcaemia*

J.M. Moseley[1], J.A. Danks[1], V. Grill[1], T.A. Lister[2] & M.A. Horton[3]

[1]*St Vincent's Institute of Medical Research, Fitzroy, Victoria 3065, Australia;* [2]*ICRF Medical Oncology Unit and* [3]*ICRF Haemopoiesis Research Group, St. Bartholomew's Hospital, London EC1A 7BE, UK.*

**Summary** The infiltrated tissues from seven West Indian patients with HTLV-1 positive adult T cell lymphoma/leukaemia (ATLL) have been analysed by immunocytochemical techniques for the presence of immunoreactive parathyroid hormone-related protein (PTHrP), a hormonal mediator of humoral hypercalcaemia of malignancy. Six of the seven were hypercalcaemic at some stage of the course of their disease. Four of the six evaluable patients showed evidence of specific cellular and extracellular expression of PTHrP protein in neoplastic tissues. This finding suggests that PTHrP may be involved in the production of hypercalcaemia in at least some cases of T cell lymphoma – proof of a causal relationship however must await the demonstration of tissue release of PTHrP resulting in raised circulating hormone levels.

Adult T cell lymphoma/leukaemia (ATLL) (reviewed in Uchiyama, 1988; Neely, 1989) is a neoplasm of lymphocytes presumptively caused by the human T cell lymphotropic virus, HTLV-1, (Yamaguchi *et al.*, 1984) and exhibiting a characteristic morphology. It has a pronounced geographical distribution originally being described in south-western Japan (Uchiyama *et al.*, 1977) and later also shown to have a particular prevalance in Caribbean emigrants to the UK and USA (Catovsky *et al.*, 1982; Blayney *et al.*, 1983; Bunn *et al.*, 1983; Swerdlow *et al.*, 1984). The disease is frequently associated with hypercalcaemia, which taken with its resistance to chemotherapy, contributes to the uniformly poor prognosis.

The aetiology of hypercalcaemia in ATLL has not been fully elucidated. Abnormalities in parathyroid hormone, prostaglandin E and vitamin D metabolism have been suggested, but investigation of a large series of cases of ATLL has failed to demonstrate significant abnormalities (Cohn *et al.*, 1987; Kiyokawa *et al.*, 1987; Fukumoto *et al.*, 1988). Recent studies have implicated a role for the hypercalcaemia-associated parathyroid hormone related protein (PTHrP) (Suva *et al.*, 1987; Mangin *et al.*, 1988) in the generation of hypercalcaemia in ATLL. Motokura *et al.* (1988) have demonstrated elevated levels of PTHrP mRNA and secretion of PTHrP from a cell line MT-2, derived from a case of ATLL. More recently, PTHrP protein has been isolated from leukaemic effusions in two patients with ATLL (Motokura *et al.*, 1989) and peripheral blood of another (Fukumoto *et al.*, 1989). It has also been reported that a dog lymphosarcoma, capable of inducing hypercalcaemia upon transplantation (Weir *et al.*, 1988), has raised PTHrP mRNA levels.

In this study we have localised PTHrP in ATLL by immunocytochemical techniques using a series of antibodies to synthetic peptides derived from the N-terminal and C-terminal segments of PTHrP.

### Materials and methods

### *Patients and biopsy material*

Seven patients with the clinical, haematological and histopathological features of ATLL syndrome were identified from records in the ICRF Medical Oncology Unit, St. Bartholomew's Hospital and reviewed by one of us (M.A.H.). The diagnostic criteria for the study included generalised lymphoid or tissue infiltration with lymphoblasts characteristic of ATLL (Uchiyama *et al.*, 1988; Neely, 1989); a leukaemic marrow and blood picture involving cells of a similar morphology; the presence of serum HTLV-1 antibodies; serum $Ca^{++}$ levels measured at least once; and biopsy material available for further study. Some of the pertinant clinical and laboratory features of the seven cases are summarised in Table I.

Standard histopathology, formalin-fixed wax-embedded, biopsy material was used for immunohistological analysis. All was archival and had not been prepared especially for the study; of note, tissue fixation times (ideally less than 24 h for detection of PTHrP) may have been prolonged, particularly with the marrow trephine biopsy samples. All available biopsy tissue was studied from each case, including multiple tissue samples from five of the patients.

### *Antibodies*

Rabbit antisera to synthetic PTHrP peptides – N-terminal PTHrP (1–34), and C-terminal PTHrP (107–141) – were prepared as previously described by us (Danks *et al.*, 1989; 1990) and used without further purification and at optimal dilutions (between 1:40 and 1:200 according to the antiserum batch used) assessed by titration on sections of normal skin and squamous carcinoma of the lung. Preimmune rabbit serum or irrelevant primary antibodies (to HIV-1 peptides) were used as negative control 'antisera'. Other controls consisted of omission of any of the other stages of the immunoperoxidase technique and inhibition of immunological reactivity of (positive) antisera by preincubation with cognate peptide ($1\ mg\ ml^{-1}$ overnight at 4°C).

### *Control tissues*

Positive control tissues were included with each run of test samples. They were either normal skin biopsies (which showed characteristic staining of the keratinocyte layer (Danks *et al.*, 1989; Hayman *et al.*, 1989)) or tissue from squamous carcinomata of the lung, which showed epithelial staining (Danks *et al.*, 1989) (results not shown). We have previously demonstrated that normal foetal lymphoid tissues (spleen, thymus (Moseley *et al.*, in press)) and lymph node from cases of non-ATLL Non-Hodgkin's lymphoma (Danks *et al.*, 1989) fail to stain with antisera to PTHrP peptides.

Correspondence: M.A. Horton.
*Presented in preliminary form at the joint 10th International Conference on Calcium Regulating Hormones and 11th Annual Meeting of the American Society for Bone and Mineral Research, Sept. 1989, Montreal, Canada. *J. Bone Min. Res.*, 4, (suppl. 1): S317 (1989).
Received 7 January 1991; and in revised form 22 May 1991.

**Table I** Carribean T-cell lymphoma/leukaemia (ATLL): patients details, calcium levels and tissue PTHrP expression

| *Patient* | *Age/Sex* | *'Lymphadenopathy'* | *'Leukaemia'* | *HTLV-1 Ab* | *Serum calcium Presentation* | *Serum calcium Maximum* | *Tissues examined*† | *PTHrP Immunoreactivity with PTHrP (1–34) antibody** |
|---|---|---|---|---|---|---|---|---|
| SG | 45/F | Cervical | No | + | 3.12 | 3.12 | Lymph node | + + + (variable) |
| MH | 49/F | Generalised | Yes | + | 2.50 | 3.35 | Lympho node (trephine) | ** |
| JT | 21/F | Generalised | Yes | + | 3.21 | 3.21 | Lymph node (trephine) | – |
| CM | 31/F | Gut lymphoma | No | + | 2.55 | 3.35 | Gut, (trephine) | – |
| FB | 48/M | Generalised | Yes | + | 3.72 | 4.64 | Lymph node (trephine) | + + |
| CP | 44/M | Generalised | No | + | 2.44 | 2.44 | Lymph node | + + + |
| PG | 40/F | Cervical, skin | No | + | 3.68 | 3.72 | Lymph node (trephine) | + (variable) |
| | | | | | [Normal range 2.20–2.67 mmol.l$^{-1}$] | | | |

†See text for comments on staining of bone marrow trephine biopsies. *Similar results obtained with antiserum to the PTHrP C-terminal peptide. **High background non-specific staining – see text.

*Immunohistological techniques*

Indirect immunoperoxidase or peroxidase-antiperoxidase (PAP) staining of dewaxed test and control tissue sections was carried out as detailed before (Danks *et al.*, 1989; Hayman *et al.*, 1989). Control antisera and tissues were included and allowed the interpretation of tissue staining (read independently by three observors) on an arbitary scale: (negative or background non-specific staining only) to + + + (strongly positive cytoplasmic and/or extracellular and connective tissue staining).

### Results

*Clinical and morphological features*

Seven patients (five female and two male West Indians of ages 21–49 years), who had the typical features of Caribbean ATLL syndrome, have been studied (see Table I). All had localised or general lymphoid organ (and in two, extranodal) infiltration and in three the disease undertook a leukaemic course. Antibodies to the HTLV-1 retrovirus were detected in serum from each case. Tissue biopsy (lymph node, skin, gut and bone marrow trephine) and marrow morphology was characteristic of ATLL (Uchiyama, 1988; Neely, 1989) showing proliferation of pleomorphic neoplastic lymphoblasts of T cell immunophenotype with typical lobulated nuclear outline (for example, see Figure 1); the clinical and immunopathological features of four of the cases have been reported previously (Swerdlow *et al.*, 1984). Hypercalcaemia was a prominent feature of this group of patients: four (57%) had hypercalcaemia at presentation (mean serum $Ca^{++}$ 3.03 mmol.l$^{-1}$) and all but one (86%) at some stage of the short clinical course of their disease (mean maximum serum $Ca^{++}$ = 3.40 mmol.l$^{-1}$ for the group).

*Immunocytochemical demonstration of PTHrP in lymphoid tissue from patients with ATLL*

No staining of patient tissue was seen if any of the immunoperoxidase reaction steps were omitted, nor if primary antibody was substituted with wash medium or an irrelevant antiserum. Immune anti-PTHrP peptide sera (to N- and C-terminal regions) reacted strongly with positive control tissues (allowing optimal antibody dilutions to be found) and failed to react with a range of tissues which did not express PTHrP, including foetal and adult lymphohaemopoietic organs (Danks *et al.*, 1989; Moseley *et al.*, in press) (data not shown). The reactivity of the antisera was blocked (although not totally) by preincubation with high concentrations of cognate peptide.

Tissue was examined from all seven patients. Of these, one (MH) gave an unacceptably high level of non-specific staining in all samples tested and the positive staining for PTHrP was considered unreliable – for this reason data is presented from the remaining six evaluable cases.

Specific cellular and/or extracellular connective tissue staining was seen using both immune antisera (maximal staining being found with the N-terminal anti-PTHrP (1–34) serum) in all infiltrated tissues from four of the patients (summarised in Table I and illustrated in Figure 1). No PTHrP was found in any of the trephine biopsies available from five of the cases, despite morphologically detectable tumour infiltration in three; it was likely that prolonged fixation and decalcification affected PTHrP immunoreactivity, a problem previously encountered.

PTHrP expression was mainly cytoplasmic in tumour cells in three of the four positive cases (F.B., C.P., P.G.) and varied from weakly (Figure 1b) to strongly positive (Figure 1g); the level of immunoreactivity to C-terminal PTHrP antisera was generally lower but proportional (see Figure 1h) to that seen with N-terminal reagents. In one case (S.G.) the distribution of PTHrP was mainly, though not exclusively, extracellular and connective tissue-associated (connective tissue staining shown in Figure 1d and area of cellular staining in Figure 1e); again this distribution was reflected, but at a lower level, with the anti-C-terminal PTHrP antiserum (not shown).

No clear correlation was found in this small group of patients between the degree or type of PTHrP staining and the level of serum $Ca^{++}$ at or after patient presentation.

### Discussion

This study demonstrates the presence of PTHrP in the neoplastic tissues of the majority of cases of HTLV-1 positive 'Caribbean' lymphoma, ATLL, a disease known to have a strong association with hypercalcaemia. All but one patient were hypercalcaemic at some stage of their disease. In four cases there was good immunological evidence for the expression of PTHrP protein. Antisera to peptides from two segments of the PTHrP protein reacted, albeit to a different extent, with tumour cells and extracellular structures in infiltrated tissues.

A variety of mechanisms have been proposed to account for the development of abnormal calcium metabolism and hypercalcaemia in ATLL. Abnormalities in parathyroid hormone, vitamin D, prostaglandin E and tumour necrosis fac-

**Figure 1** Photomicrographs of lymph node sections stained with anti-PTHrP antisera by immunoperoxidase technique. **a**, **c**, **f**: negative controls from patients (P.G., S.G., C.P.), respectively. **b**, **d**, **e**, **g**: anti-PTHrP (1–34) peptide antiserum showing: weak staining (**b**, patient P.G.), extensive extracellular and connective tissue staining with areas of cellular staining (**d** and **e**, patient S.G.) and strong intracellular expression of PTHrP (**g**, patient C.P.). **h**: section of lymph node from patient (C.P.) stained with anti-PTHrP C-terminal peptide serum (compare with **g**). (All magnified ×200 except (e), ×100).

tor α metabolism have all been proposed (Cohn *et al.*, 1987; Kiyokawa *et al.*, 1987; Fukumoto *et al.*, 1988; Adams *et al.*, 1989; Matsuda *et al.*, 1990). Recent studies have suggested a role for PTHrP, a new parathyroid hormone-like protein (Suva *et al.*, 1987; Mangin *et al.*, 1988) which is involved in normal calcium homeostasis in the foetus and implicated in the development of humoral hypercalcaemia of malignancy syndrome seen associated with epithelial tumours (Ikeda *et al.*, 1988; Danks *et al.*, 1989; Burtis *et al.*, 1990; and reviewed in Martin & Suva, 1989; Kelly & Eisman, 1989). Thus, HTLV-1 positive T cell lines and tissue from three cases of ATLL expressed PTHrP mRNA and bioactive PTHrP was isolated from cells or conditioned media (Motokura *et al.*, 1988, 1989; Fukumoto *et al.*, 1989). Taken with our immunohistochemical data we can conclude that PTHrP protein, at least in some cases of ATLL, can be synthesised by these tumours. However, there was no definite evidence in this small series for a correlation between the extent of PTHrP staining and the degree of hypercalcaemia; indeed, one PTHrP-positive case was normocalcaemic, although abnormal calcium metabolism might have still been present (Fukumoto *et al.*, 1988). Thus, a pathogenetic relationship between tumour synthesis of PTHrP and induction of hypercalcaemia remains to be proven and will await improvements in the serum assay for this hormone (Burtis *et al.*, 1990). The abnormal localisation of PTHrP in a second neoplastic tissue in association with hypercalcaemia would, though, support the contention that PTHrP is mechanistically involved in the development of hypercalcaemia in lymphoma.

We thank the Imperial Cancer Research Fund and the National Health and Medical Research Council of Australia for financial support.

### References

ADAMS, J.S., FERNANDEZ, M., GACAD, M.A. & 4 others (1989). Vitamin D metabolite-mediated hypercalcaemia and hypercalciuria patients with AIDS- and Non-AIDS-associated lymphoma. *Blood*, **73**, 235.

BLAYNEY, D.W., JAFFE, E., FISH, R.I. & 6 others (1983). The human T-cell lymphoma/leukemia virus, lymphoma, lytic bone lesions and hypercalcemia. *Ann. Intern. Med.*, **98**, 144.

BUNN, P.A., SCHECHTER, G.P., JAFFE, E. & 7 others (1983). Clinical course of retrovirus associated adult T-cell lymphoma in the United States. *N. Engl. J. Med.*, **309**, 257.

BURTIS, W.J., BRADY, T.G., ORLOFF, J.J. & 7 others (1990). Immunochemical characterization of circulating parathyroid hormone-related protein in patients with humoral hypercalcaemia of cancer. *N. Engl. J. Med.*, **322**, 1106.

CATOVSKY, D., GREAVES, M.F., ROSE, M. & 11 others (1982). Adult T-cell lymphoma leukaemia in blacks from the West Indies. *Lancet*, **i**, 639.

COHN, S.L., MOGAN, E.R. & MALLETTE, L.E. (1987). The spectrum of metabolic bone disease in lymphoblastic leukaemia. *Cancer*, **59**, 346.

DANKS, J.A., EBELING, P.R., HAYMAN, J. & 5 others (1989). Parathyroid hormone-related protein: Immunohistochemical localization in cancers and in normal skin. *J. Bone Min. Res.*, **4**, 273.

DANKS, J.A., EBELING, P.R., HAYMAN, J.A. & 7 others (1990). Immunohistochemical localization of parathyroid hormone-related protein in parathyroid adenoma and hyperplasia. *J. Pathol.*, **160**, 27.

FUKUMOTO, S., MATSUMOTO, T., IKEDA, K. & 7 others (1988). Clinical Evaluation of calcium metabolism in adult T-cell leukaemia/lymphoma. *Arch. Intern. Med.*, **148**, 921.

FUKUMOTO, S., MATSUMOTO, T., WATANABE, T., TAKAHASHI, H., MIYOSHO, I. & OGATA, E. (1989). Secretion of parathyroid hormone-like activity from human T-cell lymphotropic virus type I-infected lymphocytes. *Can. Res.*, **49**, 3849.

HAYMAN, J.A., DANKS, J.A., EBELING, P.R., MOSELEY, J.M., KEMP, B.E. & MARTIN, T.J. (1989). Expression of parathyroid hormone related protein in normal skin and in tumours of skin and skin appendages. *J. Pathol.*, **158**, 293.

HORTON, M.A., DANKS, J.A. & MOSELEY, J.M. (1989). PTHrP expression in HTLV-1-positive human T cell lymphoma: Implications for mechanism of hypercalcaemia. *J. Bone Min. Res.*, **4** (Suppl. 1), S317.

IKEDA, K., MANGIN, M., DREYER, B.E. & 7 others (1988). Identification of transcripts encoding a parathyroid hormone-like peptide in messenger RNAs from a variety of human and animal tumors associated with humoral hypercalcaemia of malignancy. *J. Clin. Invest.*, **81**, 2010.

KELLY, P.J. & EISMAN, J.A. (1989). Hypercalcaemia of malignancy. *Can. Metast. Rev.*, **8**, 23.

KIYOKAWA, T., YAMAGUCHI, K., TAKEYA, M. & 5 others (1987). Hypercalcaemia and osteoclast proliferation in adult T-cell leukaemia. *Cancer*, **59**, 1187.

MANGIN, M., WEBB, A.C., DREYER, B.E. & 9 others (1988). Identification of a cDNA encoding a parathyroid hormone-like peptide from a human tumor associated with humoral hypercalcaemia of malignancy. *Proc. Natl Acad. Sci. USA*, **85**, 597.

MARTIN, T.J. & SUVA, L.J. (1989). Parathyroid hormone-related protein in hypercalcaemia of malignancy. *Clin. Endocrinol.*, **31**, 631.

MATSUDA, K., YAMAMOTO, N., KANEKO, T., IWAHASHI, M., HASHIMOTO, S. & ARAKI, K. (1990). Hypercalcaemia and serum TNF-α in T-cell leukaemia. *Lancet*, **i**, 1032.

MOSELEY, J.M., HAYMAN, J.A., DANKS, J.A. & 4 others (1990). Immunohistochemical detection of parathyroid hormone-related protein (PTHrP) in human fetal epithelia. *J. Endocrinol. Metab.*, (in press).

MOTOKURA, T., FUKUMOTO, S., MATSUMOTO, T. & 5 others (1989). Parathyroid hormone-related protein in adult T-cell leukaemia-lymphoma. *Ann. Intern. Med.*, **111**, 484.

MOTOKURA, T., FUKUMOTO, S., TAKAHASHI, S. & 4 others (1988). Expression of parathyroid hormone-related protein in a human T-cell lymphotrophic virus type I – infected T-cell line. *Biochem. Biophys. Res. Comm.*, **154**, 1182.

NEELY, S.M. (1989). Adult T-cell leukemia-lymphoma. *Western J. Med.*, **150**, 575.

SUVA, L.J., WINSLOW, G.A., WETTENHALL, R.E.H. & 7 others (1987). Molecular cloning and expression of a novel hormone cDNA encoding a parathyroid hormone-like protein in human lung cancer cells. *Science*, **237**, 893.

SWERDLOW, S.H., HABESHAW, J.A., ROHATINER, A.Z.S., LISTER, T.A. & STANSFELD, A.G. (1984). Caribbean T-cell lymphoma-leukaemia. *Cancer*, **54**, 687.

UCHIYAMA, T. (1988). Adult T-cell leukaemia. *Blood Rev.*, **2**, 232.

UCHIYAMA, T., YODOI, J., SAGAWA, K., TAKATSUKI, K. & UCHINO, H. (1977). Adult T-cell leukemia: clinical and hematological features of 16 cases. *Blood*, **50**, 481.

WEIR, E.C., NORRDIN, R.W., MATUS, R.E. & 5 others (1988). Humoral hypercalcaemia of malignancy in canine lymphosarcoma. *Endocrinol.*, **122**, 602.

YAMAGUCHI, K., SEIKI, M., YOSHIDA, M., NISHIMURA, H., KAWANO, F. & TAKATSUKI, K. (1984). The detection of human T-cell leukaemia virus proviral DNA and its application for classification and diagnosis of T cell malignancy. *Blood*, **63**, 1235.